# The first record of the millipede genus *Streptogonopus* Attems, 1914 from Vietnam, with description of a new species (Diplopoda, Polydesmida, Paradoxosomatidae)

**DOI:** 10.3897/zookeys.601.9165

**Published:** 2016-06-29

**Authors:** Anh D. Nguyen, Kuem-Hee Jang, Ui-Wook Hwang

**Affiliations:** 1 Institute of Ecology and Biological Resources, Vietnam Academy of Science and Technology, 18, Hoangquocviet Rd., Caugiay District, Hanoi, Vietnam; 2 Institute of Phylogenomics and Evolution, and Department of Biology, Teachers College Kyungpook National University, 1005, KNU Global Plaza, 80 Daehakro, Bukgu, Deagu, 702-701, South Korea

**Keywords:** Millipede, Paradoxosomatidae, first record, new species, Vietnam

## Abstract

This paper describes a new species of the millipede genus *Streptogonopus* Attems, 1914, *Streptogonopus montanus* **sp. n.** from Vietnam, the first record of *Streptogonopus* in Vietnam. The new species is characterised by the solenophore completely sheathing the solenomere, both coiled twice, and the solenophore with a small spine at its middle. The species was found at ca. 1,800–2,100 m on Ngoc Linh Mountain. This first record for the genus in the Indochina peninsula has expanded its distributional range to the easternmost part of Southeast Asian mainland. An identification key to *Streptogonopus* species is also provided.

## Introduction

The genus *Streptogonopus* Attems, 1914 was created for three species, *Strongylosoma contortipes* Attems, 1898, *Strongylosoma jerdani* Pocock, 1892 and *Strongylosoma phipsoni* Pocock, 1892 (Attems 1914). Later, Attems (1929) specified the diagnostic characters for the genus *Streptogonopus*, and synonymised *Streptogonopus contortipes* (Attems, 1898) under *Streptogonopus phipsoni* (Pocock, 1892). Attems (1936) added a new species, *Streptogonopus nitens* Attems, 1936 from Bombay Presidency, India. Soon after that, Attems (1937) revised the genus, and listed three species, *Streptogonopus phipsoni*, *Streptogonopus jerdani* and *Streptogonopus nitens* in his major revision of the family Strongylosomidae.

Jeekel (1956) referred *Strongylosoma neglectum* Silvestri, 1895 to this genus, and described another new species, *Streptogonopus aethiopicus* Jeekel, 1956 from Eritrea. He later synonymised *Streptogonopus aethiopicus* with *Streptogonopus neglectus* (Jeekel 2004). Golovatch (2000) reported the first occurrence of the genus in Thailand with a new species, *Streptogonopus degerboelae* Golovatch, 2000. Another species, *Streptogonopus jeekeli* Golovatch, 2009, was described from China, but recently re-assigned to the genus *Hedinomorpha* Verhoeff, 1933 (Golovatch 2009, 2013). Shelley (2014) and Golovatch (2015) recently reported the occurrence of the species, *Streptogonopus phipsoni*, from Pakistan, Bangladesh and Nepal.

Currently, the genus *Streptogonopus* Attems, 1914 comprises only five valid species: *Streptogonopus neglectus* from Eritrea; *Streptogonopus phipsoni* from India, Pakistan, Bangladesh and Nepal; *Streptogonopus jerdani*, *Streptogonopus nitens*, both from India; *Streptogonopus degerboelae*, from Thailand. This work provides the first record of the genus in Vietnam with the description of a new species.

## Materials and methods

Material examined was collected from the Ngoc Linh Mountain, the second highest mountain in Vietnam (ca. 2,600 m a.s.l.). All material was preserved in ethanol 80% and is housed in the Institute of Ecology and Biological Resources (IEBR), Vietnam Academy of Science and Technology, Hanoi, Vietnam.

Gonopods were removed for morphological examination. Only the left gonopod of holotype was coated with gold for scanning electron microscopy (SEM) using an ABT 32 scanning electron microscope. Line drawings were made using an Olympus microscope SZX10. Digital images were taken using a camera *Infinity3 Lumenera* attached to a Leica M205C stereomicroscope and stacked using the software *I-Solutions*.

## Taxonomy

### Family Paradoxosomatidae Daday, 1889 Tribe Xanthodesmini Jeekel, 1968

#### Genus Streptogonopus

Taxon classificationAnimaliaPolydesmidaParadoxosomatidae

Attems, 1914

Streptogonopus Attems, 1914: 219; Attems 1929: 271; Attems 1931: 113; Attems 1936: 215-216; Attems 1937: 146-147; Jeekel 1956: 76; Jeekel 1968: 84, 111; Golovatch 2000: 218; Jeekel 2004: 15-16; Golovatch 2009: 4; Nguyen and Sierwald 2013: 1315.

##### Type species.

*Strongylosoma contortipes* Attems, 1898, by original designation.

##### Diagnosis.

Jeekel (2004) stated that the genus can be recognised by the paraterga being small or completely reduced; the gonopod femorite erect, narrow at the base and widening abruptly; demarcation between femorite and postfemoral region present; the solenomere and solenophore twisted one or two times; and the solenomere completely sheathed by solenophore.

##### Remarks.

The genus can be separated into two small groups based on the absence or presence of paraterga. The former group contains *Streptogonopus jerdani* and *Streptogonopus degerboelae*, the latter comprises *Streptogonopus neglectus*, *Streptogonopus phipsoni*, and *Streptogonopus nitens*.

#### Streptogonopus montanus sp. n.

Taxon classificationAnimaliaPolydesmidaParadoxosomatidae

http://zoobank.org/DD5ECD19-5548-4BF2-964D-E59BB69ADFE1

Figs 1 , 2 , 3

##### Material examined.

*Holotype*: 1 male (**IEBR-Myr 131H**) Vietnam, Kon Tum Prov., Ngoc Linh Mts. (107°58'30"E; 15°04'09"N), primary forest, 1,900 m a.s.l., pitfall traps, 29 March – 4 April 2006, leg. Nguyen A.D.

*Paratypes*: 1 female (**IEBR-Myr 131P**) same data as holotype; 1 male (**IEBR-Myr 145**) same locality, but 1,900–2,100 m a.s.l., 21 March–9 April 2006; 2 males, 1 juvenile (**IEBR-Myr 132**) same locality, but 1,800 m a.s.l., pitfall traps, 20–26 March 2006, all leg. Nguyen A.D.

##### Diagnosis.

This species is recognised by the gonopod femorite grooved mesally, slightly curved and expanded distally, without processes; solenophore and solenomere coiled twice, equal in length. Solenophore with a small spine at ½ its length.

##### Etymology.

“*montanus*”, an adjective to emphasise that the species has been found in a mountainous region.

##### Description.

Length 26.7–28.2 mm (male) and 30 mm (female). Width of midbody pro- and metazonae 2.3–2.5 mm (male), 2.8 mm (female) and 2.7–2.9 mm (male), 3.1 mm (female), respectively.

**Figure 1.**
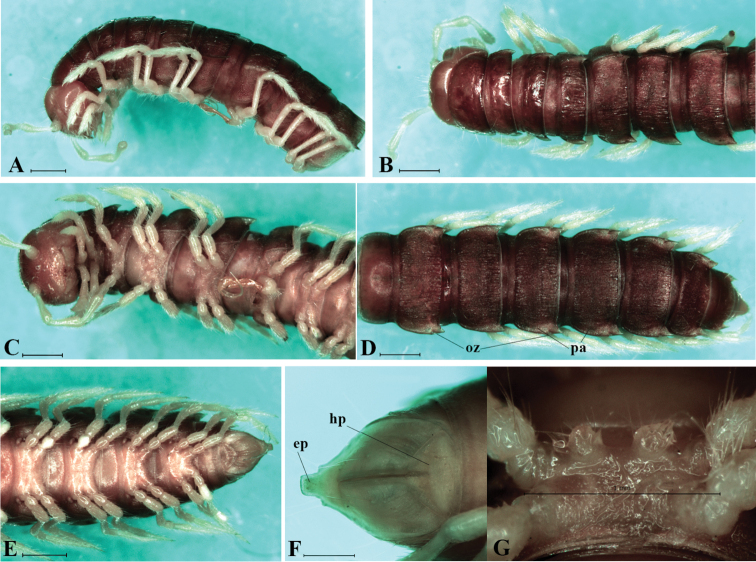
*Streptogonopus montanus* sp. n., holotype; anterior segments, lateral view (**A**), dorsal view (**B**), ventral view (**C**); posterior segments, dorsal view (**D**), ventral view (**E**); telson, ventral view (**F**); sternal processes between coxae of legpair 4, subventral view (**G**). Scale bar = 1 mm. For abbreviations see text.

Coloration (Fig. 1A–B, D): body generally castaneous or reddish brown, except legs, antenna and sterna somewhat brownish yellow.

Head subequal to collum in width; clypeolabrum modestly setose. Epicranial suture distinct. Antennae slender and long, reaching the end of segment 4 when stretched posteriorly. Antennomere 2=3=4=5=6>1>7 in length.

Collum (Fig. 1B) somewhat narrower than segment 2 in width. Surface dull and weakly wrinkled, with three rows of 4+4, 4+4 and 2+2 setae. Transverse sulcus absent. Axial line thin and evident. Paraterga modestly developed, subtriangular with broadly rounded anterior corner.

In width, segment 3<4<2=5–16, thereafter gradually tapering towards telson (Figs 1B, 1D). Prozonae shagreened. Metaterga dull, somewhat rugose with short longitudinal wrinkles, and with a row of 3+3 setae near anterior margin and a row of 3(4)+3(4) small knobs near posterior margin. Transverse sulcus starting present on metatergum 5, rather broad, not reaching base of paraterga, neither striolate nor beaded at bottom. Constriction between pro- and metazonae wide, striolate at bottom. Pleura with dense microgranulation. Pleurosternal carinae well developed on segments 2–7, reduced as a small caudal denticle on segments 8–14, then missing on subsequent segments.

Paraterga (**pa**) (Fig. 1A–B, D) modestly developed, wing-shaped, set lower than metatergal surface. Caudolateral corner pointed, spiniform on caudal segments. Paraterga surpassing posterior contour of metaterga, but not reaching next metaterga. Calluses very small, somewhat wanting on poreless paraterga, with a long seta laterally. Ozopores (**oz**) located near caudolateral corner of paraterga 5, 7, 9–10, 12–13, 15–19.

Epiproct (**ep**) (Fig. 1D, E–F) long, but broadly truncated, with four spinnerets at tip. Hypoproct (**hp**) roundly triangular, with two separated, distolateral, setiferous knobs.

Sterna (Figs 1C, 1E) moderately setose, without modifications except sternum 5 with two independent setiferous projections between coxae of legpair 4 (Fig. 1G).

Legs (Figs 1A–E) thin and slender, about 1.3 times as long as midbody height. Prefemora not swollen. Femora without modification. Tarsal brushes present only on pre-gonopodal legs.

**Figure 2.**
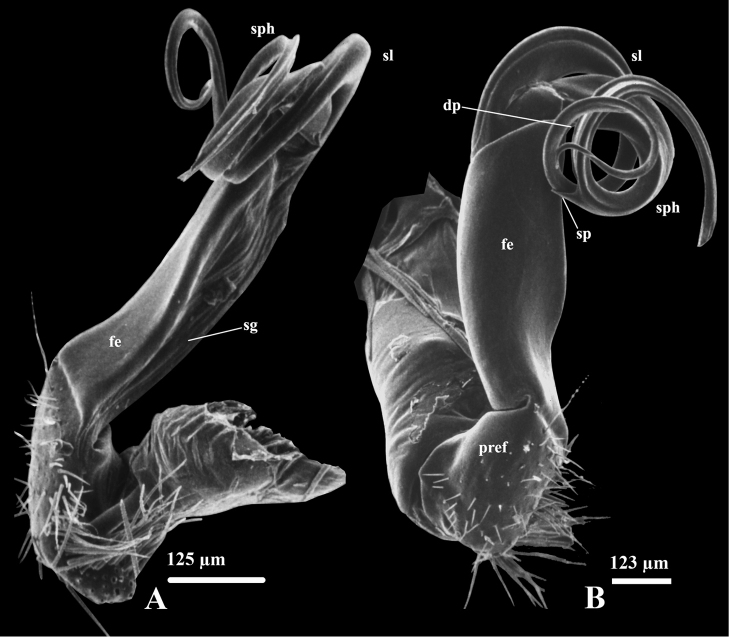
*Streptogonopus montanus* sp. n., holotype, left gonopod, mesal view (**A**), subventral view (**B**). For abbreviations see text.

**Figure 3.**
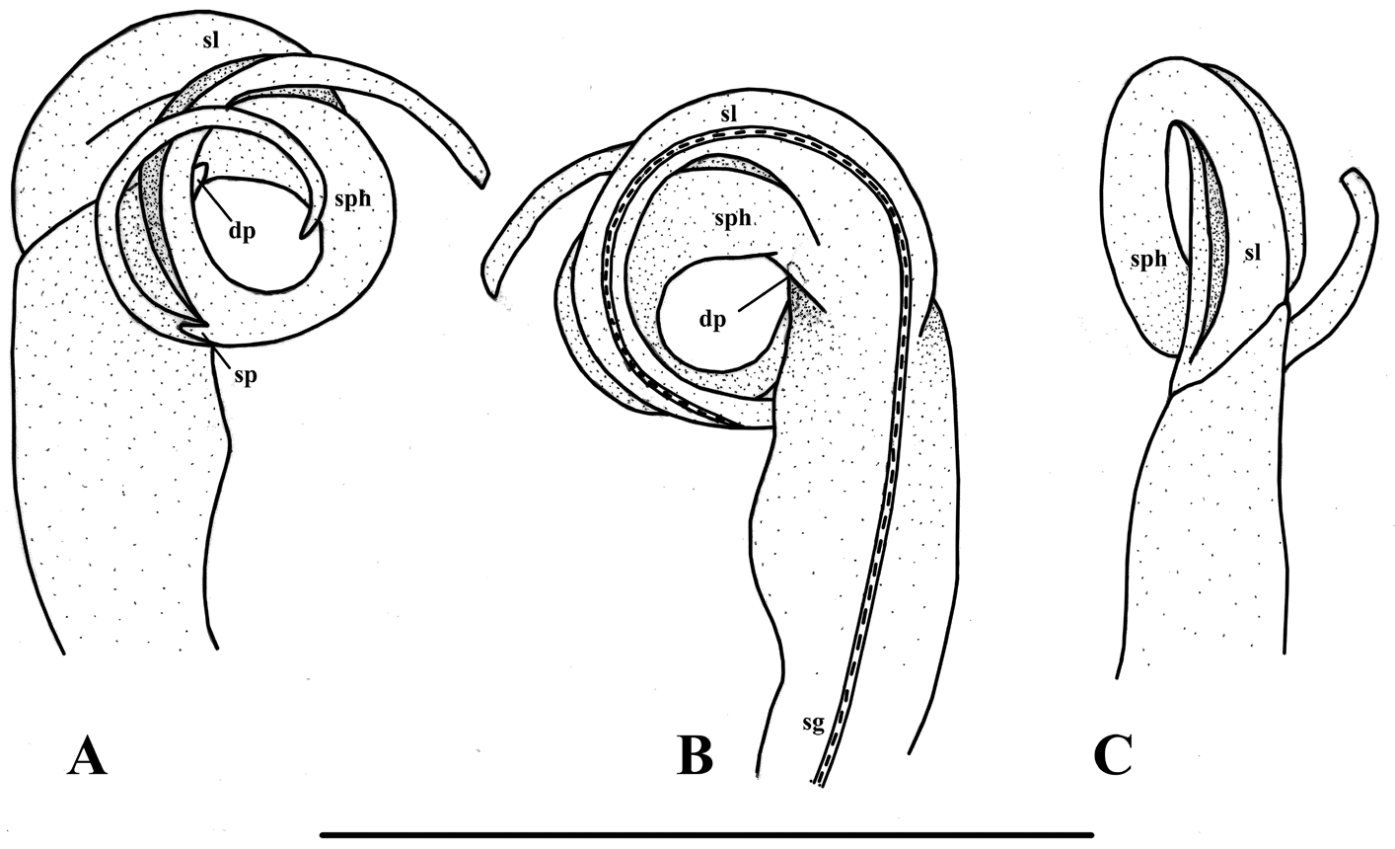
*Streptogonopus montanus* sp. n., holotype, left gonopod, ventral view (**A**), dorsal view (**B**), lateral view (**C**). Scale bar = 1 mm. For abbreviations see text.

Gonopods relatively simple (Figs 2–3). Coxite long, subcylindrical; distoventral part sparsely setose. Prefemorite (**pref**) densely setose, separated laterally from femorite by transverse sulcus. Femorite (**fe**) grooved mesally, slightly curved and expanded distad, with distolateral processes (**dp**); demarcated laterally from the postfemoral region (= solenophore) by an oblique sulcus. Solenophore (sph) and solenomere (sl) coiled more or less twice, equal in length. Solenophore with a small spine (sp) at half its length. Seminal groove (sg) running mesodorsad entirely mesally on femorite before entering the flagelliform solenomere.

##### Remarks.

This new species could be assigned to the second group characterised by modestly developed paraterga. However, it differs from three its congeners, *Streptogonopus neglectus*, *Streptogonopus phipsoni*, and *Streptogonopus nitens* in solenomere and solenophore being equal in length and strongly coiled twice and the solenophore having a small spine at its middle.

### Key to species of the genus *Streptogonopus* Attems, 1914

(based on the keys compiled by Jeekel (1956) and Attems (1936))

**Table:** 

1	Paraterga strongly reduced, even totally missing	**2**
–	Paraterga poorly to modestly developed	**3**
2	Body coloration yellowish. Body surface wrinkled and dull. India	***Streptogonopus jerdani***
–	Body coloration marble brown. Body surface smooth and somewhat shining. Thailand	***Streptogonopus degerboelae***
3	Midbody width large, more than 2 mm. Asia	**4**
–	Midbody width small, less than 2 mm. Africa (Eritrea)	***Streptogonopus neglectus***
4	Solenophore and solenomere strongly coiled twice; solenophore with a small spine at its middle. Vietnam	***Streptogonopus montanus* sp. n.**
–	Solenophore and solenomere not coiled twice; solenophore without a small spine at its middle. South Asia	**5**
5	Pleurosternal carinae caudally sharp and dentiform	***Streptogonopus phipsoni***
–	Pleurosternal carinae caudally rounded and lobiform	***Streptogonopus nitens***

## Discussion

The genus is known to occur in Eritrea, India, Pakistan, Bangladesh, Nepal and Thailand (Nguyen and Sierwald 2013; Shelley 2014; Golovatch 2015). The discovery of a new species in Vietnam has expanded the distribution range of the genus *Streptogonopus* to the easternmost part of the Southeast Asian mainland. However, there is still a gap in the distribution with no records reported from Myanmar, Laos and Cambodia (Nguyen and Sierwald 2013; Likhitrakarn et al. 2014, 2015).

The new species was found at a high elevation (ca. 1,800–2,100 m a.s.l.) on Ngoc Linh Mountain (Kon Tum Province), the second highest mountain, in Vietnam and is thus unlikely to be an introduction due to human activities. The relatively few records for the genus indicates that there are likely to be more *Streptogonopus* species awaiting discovery in Indochina and its adjacent regions.

Being located in the Burmese-Indochinese Biodiversity Hotspot (Sterling et al. 2006), Vietnam is known to have a rich fauna including millipedes. The discovery of the millipede genus *Streptogonopus* in Vietnam indicates that the Vietnamese millipede fauna may be richer at genus level than previously suspected.

## Supplementary Material

XML Treatment for Streptogonopus

XML Treatment for Streptogonopus montanus

## References

[B1] Attems C (1914) Die indo-australischen Myriopoden. Archiv für Naturgeschichte 80A(4): 1–398. http://biodiversitylibrary.org/page/46057676

[B2] Attems C (1929) Diplopoden des Belgischen Congo. I. Polydesmoidea. Revue de Zoologie et de Botanique Africaines 17(3): 253–378.

[B3] Attems C (1931) Die Familie Leptodesmidae und andere Polydesmiden. Zoologica 30(79): 1–149.

[B4] Attems C (1936) Diplopoda of India. Memoirs of the Indian Museum 11: 133–323.

[B5] Attems C (1937) Myriapoda. 3. Polydesmoidea. I. Fam. Strongylosomidae. Das Tierreich 68: 1–300.

[B6] Golovatch SI (2000) On several new or poorly-known Oriental Paradoxosomatidae (Diplopoda: Polydesmida), VII. Arthropoda Selecta 8(4): 215–220.

[B7] Golovatch SI (2009) On several new or poorly-known Oriental Paradoxosomatidae (Diplopoda: Polydesmida), VIII. Arthropoda Selecta 18(1/2): 1–7. http://kmkjournals.com/journals/AS/AS_Index_Volumes/AS_18/AS_18_1_001_007_Golovatch

[B8] Golovatch SI (2013) On several new or poorly-known Oriental Paradoxosomatidae (Diplopoda: Polydesmida), XIII. Arthropoda Selecta 22(1): 1–31. http://kmkjournals.com/journals/AS/AS_Index_Volumes/AS_22/AS_22_1_001_031_Golovatch

[B9] Golovatch SI (2015) On several new or poorly-known Oriental Paradoxosomatidae (Diplopoda: Polydesmida), XVII. Arthropoda Selecta 24(2): 127–168. http://kmkjournals.com/journals/AS/AS_Index_Volumes/AS_24/AS_24_2_127_168_Golovatch

[B10] Jeekel CAW (1956) Milliped Miscellany – Part III. Beaufortia 5(51): 73–99.

[B11] Jeekel CAW (1968) On the classification and geographical distribution of the family Paradoxosomatidae (Diplopoda, Polydesmida). Bronder-Offset Rotterdam, privately published, 162 pp.

[B12] Jeekel CAW (2004) African Paradoxosomatidae, 2: tribe Xanthodesmini (Diplopoda, Polydesmida). Myriapod Memoranda 7: 5–42. http://www.repository.naturalis.nl/document/548571

[B13] Likhitrakarn N Golovatch SI Panha S (2014) A checklist of the millipedes (Diplopoda) of Laos. Zootaxa 3754(4): 473–482. doi: 10.11646/zootaxa.3754.4.8 24869702 10.11646/zootaxa.3754.4.8

[B14] Likhitrakarn N Golovatch SI Panha S (2014) A checklist of the millipedes (Diplopoda) of Cambodia. Zootaxa 3973(1): 175–184. doi: 10.11646/zootaxa.3973.1.7 26249718 10.11646/zootaxa.3973.1.7

[B15] Nguyen AD Sierwald P (2013) A worldwide catalog of the family Paradoxosomatidae Daday, 1889 (Diplopoda: Polydesmida). CheckList 9(6): 1132–1353. doi: 10.15560/9.6.1132

[B16] Shelley RM (2014) A summary of the millipede faunas of Pakistan, Bangladesh and Kashmir (Arthropoda: Diplopoda). Insecta Mundi 0368: 1–7. http://centerforsystematicentomology.org/insectamundi/PDF-download.asp?FileName=0368_Shelley_2014.pdf

[B17] Sterling EJ Hurley MM Le DM (2006) Vietnam: a natural history. Yale University Press, 423 pp.

